# Humorous Medical Epitaphs

**Published:** 1916-12-16

**Authors:** 


### HUMOROUS MEDICAL EPITAPHS.

(FROM A CORRESPONDENT.)

Although an epitaph may present a humour that is somewhat grim, yet there is sometimes in these inscriptions drafted by relatives an (often) unconscious wit that may be appreciated. In Bath, famous for its mineral-water springs and bathing establishment, in the Abbey Church, which possesses a remarkable number of monuments upon its walls, appears on a memorial the following :—

" These walls, so full of monuments and bust,
Show how Bath waters serve to lay the dust."

Lacking the wit, but none the less candid in its criticism of mineral-water treatment, are the full text of the often misquoted words commemorating the decease of a father and his three children :—

" Here I lie with my three daughters,
Who died through drinking Cheltenham waters;
If we had stuck to Epsom salts
We shouldn't be lying in these 'ere vaults."

This suggests a method of advertising which we are glad to think the most greedy patent-medicine vendor has hitherto been unable to exploit. Another line altogether is taken by the writer of the following, who, parson though he was, and apparently a long-time sufferer, seems to have made some study of anatomy and physiology, and desired that his epitaph should form something of a popular lecture on the subject :—

" Here lies a head that often ach'd,
Here lie two hands that always shak'd,
Here lies a brain of odd conceit,
Here lies a heart that often beat;
Here lie two eyes that daily wept,
But in the night but seldom slept.
Here lies a tongue that whining talk'd,
Here lie two feet that feebly walk'd,
Here lie the midriff and the breast,
With loads of indigestion prest;
Here lies the liver full of bile,
That ne'er secreted proper chyle;
Here lie the bowels, human tripes,
Tortur'd with wind and twisting gripes;
Here lies that livid dab the spleen,
The source of life's sad tragic stream,
That left side weight that clogs the blood,
And stagnates nature's circling flood;
Here lie the nerves, so often twitch'd
With painful cramps and poignant stitch;
Here lies the back, oft rack'd with pains,
Corroding kidneys, loins and reins;
Here lies the skin per scurvy fed,
With pimples and eruptions red;
Here lies the man from top to toe,
That fabric fam'd for pain and woe;
He caught a cold, but colder death
Compressed his lungs and stopt his breath;
The organs could not longer go
Because the bellows ceas'd to blow.
Thus I dissect this honest friend,
Who ne'er till death was at wit's end;
For want of spirits here he fell;
With higher spirits let him dwell,
In future state of peace and love,
Where just men's perfect spirits move."

An event of medical interest is the theme of the lines written to commemorate Mrs. Vanbutchel, wife of the famous doctor, who endeavoured, with the help of Dr. William Hunter, so to preserve his wife's body as to give it the appearance of life.

" Here unentomb'd, Vanbutchel's consort* lies,
To feed her husband's grief or charm his eyes;
Faultless and pure her body still remains,
And all its former elegance retains;
Long had disease been preying on her charms,
Till slow she shrunk in Death's expecting arms;
When Hunter's skill in spite of nature's laws,
Her beauties rescued from corruption's jaws;
Bade the pale roses of her cheeks revive,
And her shrunk features seem again to live :
—Hunter, who first conceived the happy thought,
And here at length to full perfection brought.
O lucky husband ! blest of heaven,
To thee the privilege is given
A much lov'd wife at home to keep,
Caress, touch, talk to, even sleep
Close by her side, whene'er you will,
As quiet as if living still.
And, strange to tell, that fairer she,
And sweeter than alive should be;
Fair, plump, and juicy as before,
And full as tractable, or more.
Thrice happy mortal ! Envied lot !
What a rare treasure hast thou got;
Who to a woman can lay claim,
Whose temper's every day the same."

A gross injustice to the medical profession and a feeling approaching to anger among his patients might have arisen from the lines written on the death of a certain Dr. I. Lettsom :

" When people's ill they come to I,
I physics, bleeds, and sweats 'em;
Sometimes they live, sometimes they die;
What's that to I? I. Lettsom" (let's 'em).

The above examples only serve to remind us how difficult it is to write a good epitaph. To be truthful yet terse, to record affection without sentimentality, and to express a man's virtues without cant is a task beyond most authors. Epitaphs are generally the sole essay of the amateur, and, as in the case of most amateur efforts, he (or she) has chosen in the epitaph the one task almost sure to be beyond him. Therefore, though of no medical interest, it is perhaps allowable to conclude this article with an epitaph to an imaginary person, which was introduced by the great evolutionary writer Samuel Butler into his masterly "Erewhon Revisited." It runs : " I fall asleep in the full and certain hope that my slumber shall not be broken; and that though I be all-forgetting, yet shall I not be all-forgotten, but continue that life in the thoughts and deeds of those I loved, into which, while the power to strive was yet vouchsafed to me, I fondly strove to enter." But might we not all take with advantage Hamlet's cue, and simply say, as he did of himself, "the rest is silence"?

* The body of Mrs. Van Butchell is still preserved in the museum of the Royal College of Surgeons.—B. R.

